# Stroke secondary prevention, a non-surgical and non-pharmacological consensus definition: results of a Delphi study

**DOI:** 10.1186/s13104-019-4857-0

**Published:** 2019-12-23

**Authors:** Maggie Lawrence, Eric Asaba, Elaine Duncan, Marie Elf, Gunilla Eriksson, James Faulkner, Susanne Guidetti, Birgitta Johansson, Christina Kruuse, Danielle Lambrick, Caitlin Longman, Lena von Koch, Xu Wang, Olive Lennon

**Affiliations:** 1 Department of Nursing and Community Health, School of Health and Life Sciences, Glasgow Caledonian University, Glasgow, G4 0BA UK; 2 Department of Neurobiology, Care Sciences, and Society (NVS), Division of Occupational Therapy, Karolinska Institutet, Stockholm, Sweden; 3 Department of Psychology, School of Health and Life Sciences, Glasgow Caledonian University, Glasgow, UK; 4 School of Education, Health and Social Studies, Dalarna University, Falun, Sweden; 5 Architecture and Civil Engineering, Department of Building Design, Chalmers University of Technology, Gothenburg, Sweden; 6 Department of Neuroscience, Rehabilitation Medicine, Uppsala University, Uppsala, Sweden; 7 Department of Sport, Exercise and Health, University of Winchester, Winchester, UK; 8 Institute of Neuroscience and Physiology, Department of Clinical Neuroscience, University of Gothenburg, Gothenburg, Sweden; 9 Department of Neurology Stroke Unit and Neurovascular Research Unit, Herlev-Gentofte Hospital, Copenhagen, Denmark; 10 School of Health Sciences, Faculty of Environmental and Life Sciences, University of Southampton, Southampton, UK; 11 Stroke Association, London, UK; 12 Department of Neurobiology, Care Sciences and Society, Karolinska Institutet, Stockholm, Sweden; 13 Leeds School of Social Sciences, Leeds Beckett University, Leeds, UK; 14 School of Public Health, Physiotherapy and Sports Science, University College Dublin, Dublin, Ireland

**Keywords:** Stroke, Secondary prevention, Delphi

## Abstract

**Objective:**

Evidence supporting lifestyle modification in vascular risk reduction is limited, drawn largely from primary prevention studies. To advance the evidence base for non-pharmacological and non-surgical stroke secondary prevention (SSP), empirical research is needed, informed by a consensus-derived definition of SSP. To date, no such definition has been published. We used Delphi methods to generate an evidence-based definition of non-pharmacological and non-surgical SSP.

**Results:**

The 16 participants were members of INSsPiRE (International Network of Stroke Secondary Prevention Researchers), a multidisciplinary group of trialists, academics and clinicians. The Elicitation stage identified 49 key elements, grouped into 3 overarching domains: Risk factors, Education, and Theory before being subjected to iterative stages of elicitation, ranking, discussion, and anonymous voting. In the Action stage, following an experience-based engagement with key stakeholders, a consensus-derived definition, complementing current pharmacological and surgical SSP pathways, was finalised: Non-pharmacological and non-surgical stroke secondary prevention supports and improves long-term health and well-being in everyday life and reduces the risk of another stroke, by drawing from a spectrum of theoretically informed interventions and educational strategies. Interventions to self-manage modifiable lifestyle risk factors are contextualized and individualized to the capacities, needs, and personally meaningful priorities of individuals with stroke and their families.

## Introduction

International best practice guidelines for stroke secondary prevention (SSP), while aetiology dependent, generally include medication prescription (anti-hypertensive, lipid lowering, anti-platelet/coagulant); high level evidence supports this recommendation [1, 2]. Conversely, recommendations for lifestyle modifications have lower levels of evidence, largely drawn from primary prevention studies, and as a results some population-attributable stroke risk factors (e.g. psychosocial stress) [3] are inadequately addressed [1, 2].

Conclusive evidence is lacking on how best to support stroke survivors to engage in risk reducing behaviours. In recent systematic reviews of complex interventions in SSP, meta-analysis was possible for limited outcomes due to primary study heterogeneity across key definitions, population and intervention characteristics, outcomes and associated measures [4, 5]. To advance the evidence-base, empirical research is needed, informed by a consensus-derived definition of non-pharmacological and non-surgical SSP and an agreed core set of outcomes. No published consensus on these foundational tools exists.

In 2016, INSsPiRE (International Network of SSP Researchers), comprising secondary prevention trialists identified by ML’s reviews [4, 6] agreed a programme of work, focusing initially on a consensus-derived definition of secondary prevention, beyond pharmacological and surgical interventions, to inform research standards, facilitate data synthesis, guideline development and service delivery.

## Main text

### Methods

**Fig. 1:**
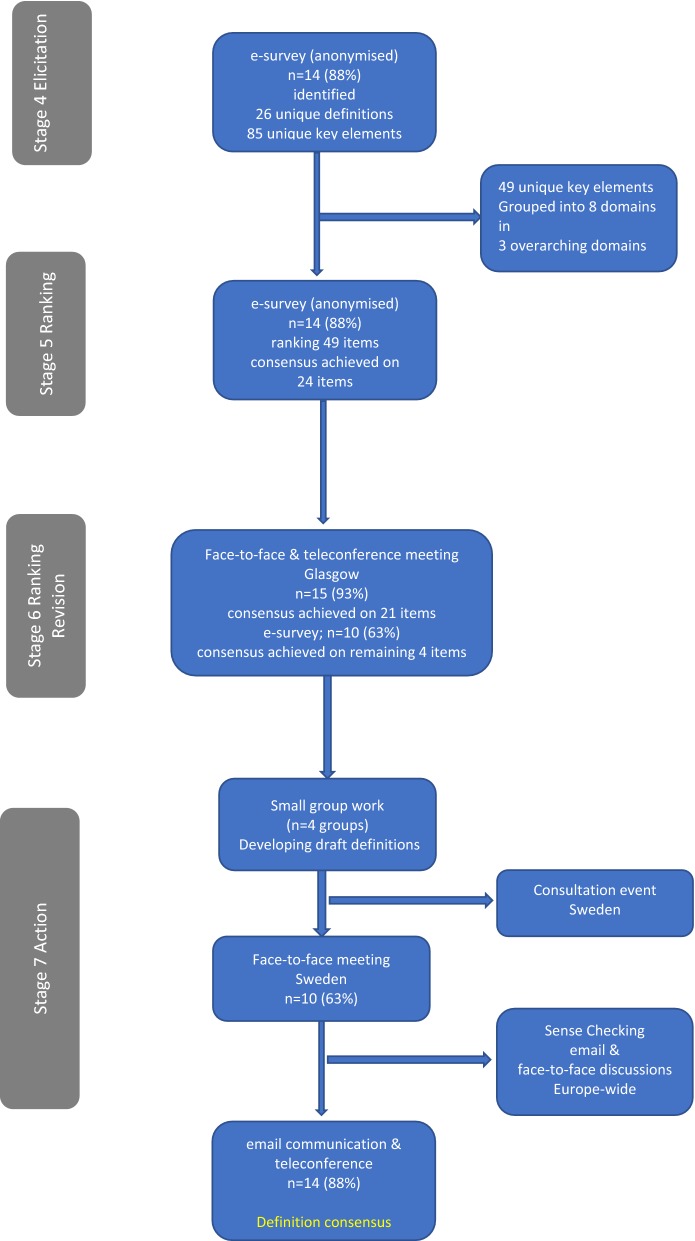
Consensus process

Delphi technique: a structured, iterative process that pools knowledge and understanding from a range of experts to arrive at an agreed standpoint on an issue [7]. Data can be gathered using electronic, internet-mediated tools; ideal where geographical barriers exist. Data were collected and synthesized in seven stages [7], using both online and in-person modes (Fig. 1).

- Stages 1 *facilitator assignment* and 2 *participant identification*: ML facilitated stages 1–4. Stage 6 was facilitated by BD, and stage 7 by AHP; independent, experienced researchers. Participants were identified as the contemporaneous members of INSsPiRE i.e. published researchers, academics, and clinicians working in the specialty of non-pharmacological and non-surgical SSP.
- *Stage 3: Problem definition:* The problem was defined as the lack of an evidence-based, consensual definition of non-pharmacological and non-surgical SSP.
- *Stage 4: Elicitation of definitions and key elements of SSP:* Participants were sent an elicitation survey by URL link. Comprising two parts, the survey asked participants to:
  1. Provide examples of published definitions of non-pharmacological and non-surgical SSP from systematic reviews, research studies, clinical guidelines, or third sector websites, including source reference(s).
  2. List key intervention elements and/or underpinning concepts and theories considered essential to non-pharmacological and non-surgical SSP.
- *Stage 5: Ranking:* Participants received a link to a survey where they ranked, *without consultation* with other participants, each key element and concept previously identified, on a 10-point Likert scale: Not important (1–3), Important but not critical (4–6), Critical (7–9); Unable to score (10).
- *Stage 6*: *Ranking revision:* This iterative stage used online and in-person modes. Participants accessed the Stage 6 survey, developed by augmenting the stage 5 survey with the item-by-item response data (i.e. voting frequency). Participants reviewed overall response rates and considered whether to revise their original ranking. Another round of anonymous ranking followed group-based discussions. Consensus was defined as ≥ 70% ranking agreement [8].
- *Stage 7: Action:* In small groups participants worked (phone/Skype/in-person) to draft definitions. At a subsequent in-person meeting, following experience-based stakeholder engagement, participants reviewed Stage 6 results and draft definitions, and discussed and agreed a definition.

Ethical approval was received from Glasgow Caledonian University’s (GCU) School of Health and Life Sciences Ethics Committee (HLS/NCH/16/020).

### Results

Table 1 identifies INSsPiRE members who participated in ≥ 1 Delphi process stage.

**Table 1:** Participant characteristics (profession, country)

Profession	Number	Country
Dietician	1	Scotland
Healthcare architect (nurse)	1	Sweden
Information scientist (nurse)	1	Scotland
Nurse	1	Denmark
Occupational therapist	3	Sweden
Physician	1	Denmark
Physiotherapist	2	Ireland/Sweden
Psychologist	2	Sweden/England
Speech and language therapist	1	South Africa
Sport and exercise physiologist	2	England
Sport and exercise psychologist	1	Scotland

In Stage 4 (Elicitation), 14(88%) participants identified 26 unique definitions of SSP and 85 unique ‘key elements’. XW collated the definitions, removed duplicates, and shared the resultant list with participants. ML collated ‘key elements’, removed duplicates, and categorised elements into eight domains: Modifiable lifestyle risk factors, Modifiable physiological risk factors, Education about stroke, Education about modifiable risk factors, Education about managing other lifestyle issues, Education about managing psychosocial factors, Skills education/training, and Underpinning theories and approaches. The domains were collapsed into three overarching domains: Risk factors, Education, and Theory, and used to structure the Stage 5 survey. In Stage 5 14(88%) participants ranked each element using the Likert scale (above).

In Stage 6 (Ranking revision) online consensus (n = 14, 88%) was achieved to include 24 of the 49 elements. At a subsequent, independently facilitated in-person meeting (n = 14, 88%) at GCU in June 2017, discussion and debate was followed by anonymous voting on the remaining 25 elements. Three elements were merged with others, one was removed, and consensus to include 14 and exclude three further elements achieved. Four outstanding elements remained; after two further online-rounds (n = 15, 94%; n = 10, 67%), consensus was achieved to include all four. By Stage 6 conclusion, 42 key elements were agreed (Table 2).

**Table 2:** Key elements of SSP: status in Stages 5 and 6

Key elements	Risk factors	Education	Underpinning theory/approaches
Consented for inclusion—Stage 5 (n = 24)	Physical inactivity, diet, current smoking, hypertension/blood pressure, cholesterol/blood lipid	Stroke risk factors, signs and symptoms of stroke, action to take if stroke is suspected, importance of adhering to medication prescription, physical activity, diet, smoking cessation, alcohol consumption, stress management, weight management, diabetes management, blood pressure management, medication adherence, emotional health, perceived psychosocial stress, self-management, self-efficacy	Psychological theories of wellbeing; patient-centred/person-centredness
Consented for inclusion—Stage 6a (n = 14)	Alcohol consumption, psychosocial factors	Prescription medications for stroke, work/life balance, anxiety, depression, goal setting, pacing, establishing networks, self-monitoring	Behaviour change, implementation theory, self-management, ‘family’-centredness (caveat: definition of ‘family’ be explained, or terminology changed to represent its inclusive nature)
Consented for exclusion—Stage 6a (n = 3)	Waist/hip ratio, blood sugar		Family theory e.g. Calgary family assessment and Intervention model
Elements merged/removed after Stage 6a (n = 4)		Exercise counselling (*removed*); stroke education (*merged* with ‘what is stroke’); goal prioritising (*merged with* ‘goal setting’	Cognitive and emotional models for modification (*merged* with ‘Behaviour change theories’ to form ‘Cognitive, emotional and behaviour change models’)
Consented for inclusion—Stage 6b (n = 2)		What is stroke, problem solving	
Consented for inclusion—Stage 6c (n = 2)		Sleep, opportunities to practice new skills	

In Stage 7 (Action), participants (n = 15, 94%) worked in small groups to draft definitions, which were ranked in a subsequent online round; no consensus was achieved. In September 2018, prior to an in-person Delphi meeting, participants (n = 10, 67%) met with experience-based stakeholders at Karolinska Institutet, Stockholm. This consultation enabled Delphi participants to consider the relevance and meaningfulness of agreed key elements and draft definitions from stakeholders’ perspectives. At the Delphi meeting, a final consensus-definition was agreed and subsequently sense-checked and finalised electronically by all participants: *Non*-*pharmacological and non*-*surgical stroke secondary prevention supports and improves long*-*term health and well*-*being in everyday life and reduces the risk of another stroke, by drawing from a spectrum of theoretically informed interventions and educational strategies. Interventions to self*-*manage modifiable lifestyle risk factors are contextualized and individualized to the capacities, needs, and personally meaningful priorities of individuals with stroke and their families.*

## Discussion

This consensus-driven definition moves the concept of non-pharmacological/non-surgical SSP forward. Previous ambiguity around SSP meant inherent difficulty in formulating appropriate research questions, standardising outcome measures, and synthesising evidence.

Non-pharmacological/non-surgical SSP, as defined, is not intended to stand-alone as a preventive strategy. Rather, it raises awareness of additional avenues for focus to maximise reduction in recurrent events. Gains from modest lifestyle changes in addition to pharmacological interventions have an estimated cumulative relative risk reduction for recurrent events of 80% (Numbers Needed to Treat: 5) [9]; notably pharmacological adherence is a health behaviour core to our definition [10].

Reaching consensus by electronic voting alone proved challenging for a number of ‘key elements’ including unsafe alcohol consumption, and psychosocial stress, both of which receive little attention in SSP RCTs [4, 5, 11], and addressing psychosocial stress is not an SSP guideline recommendation [1, 2]. Similarly, insufficient evidence exists to recommend any one behaviour change and/or self-management theory in SSP. When achieving consensus became protracted, face-to-face meetings allowed effective open debate prior to anonymous voting.

Future work must include agreement on core outcomes for non-pharmacological, non-surgical SSP. Informed by this definition, a planned overview review will determine the quantity and quality of evidence from theoretically-informed studies employing behavioural and/or self-management strategies on mortality, cardiovascular end points, and risk-reducing behaviours [12]. The consensus definition presented here is an important first step in building an impactful, evidence-based field in SSP.

## Limitations

- Small, self-selecting, sample
- Limited representation of healthcare cultures and infrastructures outside of north-west European.

## Data Availability

The dataset(s) supporting the conclusions of this article are available in the Open Science Forum repository: osf.io/r5wdg/?view_only = 4e84c9dcf0064457b4bac422f341c546.

## Abbreviations

GCU: Glasgow Caledonian University
SSP: stroke secondary prevention
INSsPiRE: International Network of Stroke Secondary Prevention: Researchers
URL: uniform resource locator
RCT: randomised controlled trial
